# Effects of Motor Intervention Program on Academic Skills, Motor Skills and Social Skills in Children with Autism Spectrum Disorder

**DOI:** 10.1007/s10803-024-06384-5

**Published:** 2024-05-16

**Authors:** Gülsüm Hatipoğlu Özcan, Dilara Fatoş Özer, Salih Pınar

**Affiliations:** 1 Faculty of Sport Sciences, Istanbul Yeni Yüzyıl University, Istanbul, Turkey; 2 Department of Child Development, Faculty of Health Sciences, Istanbul Bilgi University, Istanbul, Turkey; 3 Faculty of Sport Sciences, Fenerbahçe University, Istanbul, Turkey

**Keywords:** Autism spectrum disorder, Motor skills, Academic skills, Social skills

## Abstract

The aim of this study was to examine the effect of motor intervention program (MIP) on autistic index, pre-academic skills, motor skills and social skills of children with Autism Spectrum Disorder (ASD). The research group consisted of a total of 34 participants between the ages of 3–6, 17 in the control group (CG) and 17 in the experimental group (EG). EG participated in the motor intervention program for 60 min a day, 2 days a week for 12 weeks. In the study, the Gilliam Autistic Disorder Rating Scale-2-Turkish Version (GARS-2 TV), Peabody Motor Development Scale-2 (PMDS-2), Pre-Academic Skills Evaluation Form (PASAF) and Social Skills Evaluation System Preschool Teacher Form (SSRS-PTF) were used. The increase in all subtests and total scores of PASAF and posttest scores obtained from PMDS-2 were found to be higher in favor of the experimental group (p < 0.05). The decrease in the stereotype and social interaction scores of GARS-2 TV and the change in the cooperation, self-control and externalization sub-dimensions of SSRS-PTF were found to be statistically significant in favor of the EG group (p < 0.05). In conclusion, it was found that MIP applied to autistic children was effective on the development of motor skills, academic skills and social skills and decreased the level of autistic index. This result shows that MIP is an effective practice that provides a favorable environment for autistic young children to develop multiple skills.

Autism Spectrum Disorder (ASD) is a neurodevelopmental disability that typically manifests as impaired social communication and restricted, repetitive behaviors in early childhood and ranges from mild to severe (American Psychiatric Association [APA], 2013). According to the prevalence rates determined by the Centers for Disease Control and Prevention (CDC) Autism and Developmental Disabilities Monitoring (ADDM) network, ASD, which was seen in approximately one in 150 children in 2000, was found to be seen in 1 in 36 children in 2020 and was reported to be 4 times more common in boys than in girls. This shows that the rate of autistic children increased by 2.11% in 2020 compared to 2000.

In addition to the social and emotional developmental characteristics specified in the Diagnostic and Statistical Manualof Mental Disorders-5 (DSM-5) criteria (APA, 2013), delays and impairments in motor development began to be documented by some researchers in the 1990s (Teitelbaum et al., 1998) and became the main subject of numerous studies, especially since the beginning of 2000 (Ozonoff et al., 2008). These studies indicate that the motor skills of autistic children are qualitatively weak and that these children are delayed in terms of fine and gross motor skill development compared to their typically developing peers of the same age (Bremer et al., 2015; Busti Ceccarelli et al., 2020; Liu, 2012; Liu & Breslin, 2013; Lloyd et al., 2013; McDonald et al., 2014; Odeh et al., 2022; Oliveira et al., 2021; Özcan et al., 2023; Zampella et al., 2021).

Furthermore, an increasing body of literature provides evidence that these delays commence early (Liu, 2012; Özcan et al., 2023; Ozonoff et al., 2008; Posar & Visconti, 2022), and demonstrates that early motor difficulties, which may manifest even before the onset of core autism features in autistic children (Posar & Visconti, 2022), and motor delays are now the norm rather than the exception (Bhat et al., 2011; Setoh et al., 2017; Zampella et al., 2021). In a study conducted on a large sample group of children younger than 6 years of age on the autism spectrum, it was reported that motor difficulties in autistic children were common and the need to consider motor difficulties more as a separate determinant in the diagnostic criteria of ASD was emphasized (Licari et al., 2020).

As the frequency with which the social deficits that characterize ASD, such as joint attention, social orientation, delay in acquiring imitation and play skills, and atypical development, are identified with ASD increases, the need for effective strategies targeting the development of social skills also increases (Scattone, 2007). Innovative experimental methodologies and theoretical studies of social development have yielded important findings over time, contributing to our understanding of both typical and atypical developmental features in ASD (Charman & Stone, 2008). Some of these studies show that gross motor difficulty is associated with the basic social deficits that characterize autism (Derer, 2018; Ohara et al., 2019; Wang et al., 2022) and that the social communication deficits exhibited by children on the autism spectrum can be supported through motor programs (Chu & Pan, 2012; Elliott et al., 2021; Ketcheson et al., 2017; McLaughin, 2010; Parlak, 2021; Zampella et al., 2021).

Research focusing on increasing the cognitive development and academic performance of children on the autism spectrum provides evidence that physical activity has a positive effect on executive functions, attention development and academic performance (De Greeff et al., 2018; Tan et al., 2016), motor skills are associated with non-verbal IQ, and children with poorer motor skills have more cognitive and adaptive behavior deficits (Fulceri et al., 2015). Therefore, it is suggested that motor deficits seen in the autism spectrum are associated with poor cognitive performance and problem behaviors (Piek et al., 2008).

On the other hand, the results of the limited number of studies examining the effects of motor programs on the characteristics of ASD show that there is a direct relationship between the level of ASD and the level of motor skills (Sedehi et al., 2021; MacDonald et al., 2014; Hilton et al., 2007). In addition, studies report that supporting children on the autism spectrum through motor programs has positive effects in terms of reducing stereotypical behaviors, increasing appropriate responses, making eye contact, and social interaction as well as improving motor skills (Chu & Pan, 2012; Keskin et al., 2017; Kruger et al., 2019; McLaughin, 2010; Obrusnikova & Miccinello, 2012; Prupas & Reid, 2001; Öz, 2021; Richmond, 2000).

Although development is examined under three main areas: cognitive, affective and motor, when children's behaviors are examined, it is seen that development is actually a whole and motor development and other developmental areas are interrelated (Goodway et al., 2019). Unfortunately, parents tend to focus on the more pressing issues raised by the diagnosis and place motor coordination issues lower on their priority list (Kurtz, 2007). Moreover, the motor skills of young autistic children are not even seen as a priority for early interventionists, who focus primarily on communication and behavioral concerns (Lloyd et al., 2013).

Existing research points out that young children on the autism spectrum have significant motor delays and that delays become more pronounced with age (Busti Ceccarelli et al., 2020; Landa & Garrett-Mayer, 2006; Lloyd et al., 2013; Odeh et al., 2022; Oliveira et al., 2021; Özcan et al., 2023; Zampella et al., 2021). Despite this, it is seen that movement-based activities are neglected in the educational process in the preschool period (Elias, 1997), studies on the motor skills of children on the autism spectrum in early childhood are insufficient (Colombo-Dougovita & Block, 2019; Ketcheson et al., 2017; Ozonoff et al., 2008), and most of the studies on individuals on the autism spectrum focus on children older than 6 years (Görgün & Melekoğlu, 2016). However, early childhood years have an importance that prepares the ground for the later periods of an individual's life and can have a positive or negative impact on these periods (Özer & Özer, 2012). Moreover, the period of basic movements, which covers these years, is a critical period in which the basic movements necessary for life to continue independently are acquired. In addition, basic motor skills are important because they play a key factor in physical activities, sports and general life functioning (Goodway et al., 2019).

In the light of the literature mentioned above, this study aimed to investigate the effects of motor intervention program, which was prepared based on motor skills and aimed to develop academic skills and social skills with a holistic approach, on motor skills, academic skills, social skills and autistic index.

## Methods

### Research Model

In this study, a true experimental design with pretest–posttest control group was used. In real experimental models, groups are formed through random assignment and these groups are considered equal in terms of control variables (Büyüköztürk et al., 2008). Participants in this study were randomly assigned to the control and experimental groups using Microsoft excel program. After the pre-test measurements were completed, it was determined that there was no significant difference between the two groups, thus both groups had similar characteristics before the motor intervention program (MIP).

### Participants

This study was conducted with a total of 34 autistic children aged between 3 and 6 years, 17 in the experimental group (EG) and 17 in the control group (CG), who were receiving education in 4 different special education and rehabilitation centers in Istanbul. The inclusion criteria were that the participants were between the ages of 3–6 years, had not participated in a MIP before, did not have a health problem that prevented participation in the MIP, were diagnosed with autism spectrum disorder (ASD), and did not have a secondary diagnosis such as visual or hearing difficulties. The exclusion criteria for the participants were as follows; for EG, having a health problem that prevented them from participating in the MIP, EG and CG not participating in the tests, and participants voluntarily leaving the process for any reason during the trainings and measurements.

### Measures

At the end of each session of the MIP, the assessments made through observation were noted on an observation tracking list for each child. This list was created by the research team. The list included items such as the child's interest in the training session, performance towards target behaviors, level of following instructions and the reasons for the emergence of problem behaviors. This enabled us to identify the priority goals that each child needs to be supported with and relevant educational strategies for the next session.

### Procedures

The study started with the data collection process for the pretests after obtaining the approval of the ethics committee numbered 09.2020.920, parental consent approval from the parents of the participants, and written consent from the institutions where the participants were studying that they would support the study. The tests and MIP applied during the research process were conducted at the institutions where the participants were studying.

The administration time of the Peabody motor development scale 2 (PDMS-2) for each child was 25–30 min, while the Pre-academic skills assessment form (PASAF) was conducted in 10–15 min. While answering the Social Skills Rating System Preschool Teacher Form (SSRS-PTF) by the special education teacher of the autistic children required approximately 10 min for each child, the GARS-2 TV, which was administered through a face-to-face interview with the parent of the autistic children, was completed in 25–30 min. The certificate of use required to administer the GARS-2 TV scale was obtained by the first author of the study after attending the training.

During MIP, innovative ASD-specific teaching strategies and methods such as using activity charts and visual cues, giving instructions in clear and short sentences (Grenier, 2013), allowing children to make choices, providing concrete experiences, and modeling (Özer et al., 2020) were used. Thus, effective pedagogical approaches were taken as a basis for the teaching processes of the skills targeted for the participants. In addition, at the end of each training session, the research team and the special education teacher made an individual assessment of the child's interest and performance in the activities on a form.

### Motor Intervention Program

The MIP was prepared by the researchers for three developmental domains (motor skills, pre-academic skills and social skills) based on the pre-test items of the data collection tools. MIP consists of activities that aim to develop motor skills (throwing, catching, running, jumping, etc.) as well as pre-academic skills (geometric shapes, colors and numbers) and social skills (greeting, waiting for one's turn, making friends, cooperating, etc.). In other words, MIP is designed as a program that focuses on other areas of development of the child through activities based on basic movement skills.

The program was implemented for 60 min a day, 2 days a week for 12 weeks in the institutions where the children were studying by the first author of the study. Considering the results obtained from the pre-test evaluations, 25% of the training session for the skills that the participants needed to be supported individually was implemented as individual activities and 75% (45 min.) as group activities that allowed all children to interact with each other. Thus, both individual activities and group activities (more intensively) were carried out within the same training session. The field implementations of the education program were carried out in the presence of the research team and 8 special education teachers working in the special education and rehabilitation institutions that the children attended. In this way, at least one teacher supported each child during the implementations.

The MIP activities were implemented in four phases, similar to the preparation of a physical education activity plan for preschool. These phases were as follows: “(i) immersive movements consisting of warm-up movements such as walking, running and jumping (5 min), (ii) functional exercises that improve joint mobility and flexibility to prepare the body to practice the targeted skills (5 min), (iii) individual and group activities focusing on 3 developmental areas (motor skills, social skills and pre-academic skills) based on basic movement skills (40 min), and (iv) whole group activities consisting of games that reinforce skills for the targeted developmental areas and increase interaction (10 min).

### Data Collection Tools

#### Gilliam Autistic Disorder Rating Scale-2-TV

Turkish adaptation of the Gilliam Autistic Disorder Rating Scale-2, which was developed by Gilliam (2006) to determine and rate the autistic disorder indices of children with autistic disorder, was conducted by Diken et al., (2012). The GARS-2-TV includes three subscales, namely Stereotypical Behaviors, Communication and Social Interaction, and consists of a total of 42 items, with 14 items in each subscale. GARS-2-TV, which is a 4-point Likert-type scale (0 = not observed, 1 = rarely observed, 2 = sometimes observed, 3 = frequently observed), is evaluated in the range of highest 153 standard points and lowest 55 standard points. The higher the standard score, the higher the probability of autistic disorder (Diken et al., 2012).

#### Peabody Motor Development Scale-2

The Peabody Motor Development Scale (PMDS) was revised by Folio and Fewell in 2000 and renamed as PMDS-2. The PMDS-2, which was developed to determine the gross and fine motor development levels of children aged 0–6 years, has been accepted as a valid scale to identify delays and disorders in the motor development of preschool children. The scale consists of two dimensions (gross and fine motor) and six sub-dimensions with a total of 249 items. PDMS-2 norms require scoring each item as 2, 1 or 0 according to the child's level of realization of the item with the desired quality. When 2 points are obtained from three consecutive items in the scale, the base level is determined, and when 0 points are obtained, the ceiling level is determined. While all items above the base level are considered as 2 points, all items below the ceiling level are recorded as 0 points and the application of the scale is terminated (Folio & Fewel, 2000).

#### Pre-Academic Skills Assessment Form

The Pre-academic skills assessment form (PASAF) created by Özcan (2022) is an assessment test that can be applied to both typically developing children and children with special needs between the ages of 3 and 6. The basic concepts in this form, which are determined in line with preschool education goals, are grouped under the titles of color, geometric shape, dimension and numbers and consist of 4 subtests and 34 items. The PASAF criteria are based on scoring each item as 1 or 0. A score of 1 means that the child answered the item correctly; a score of 0 means that the child did not answer the item correctly or did not respond to the item. The number of correct answers obtained from each subtest constitutes the total subtest score and the sum of all subtests is recorded as the overall score.

The content validity ratio (KVR) of the items of the PASAF created within the scope of this study was evaluated in line with the expert opinions of 5 academicians. The scores obtained from the evaluations were calculated according to the formula KVRi = (NG − (N/2)) developed by Lawshe (1975) and most widely used for content validity. According to this formula, KVRi takes a value between − 1.0 and 1.0. The 5 experts who participated in the study expressed their opinions as "necessary" for 34 items in the form. The KVRi values of all items in the form were calculated as [(5–2.5)/2.5] = 1.00 and the content validity was found to be high.

#### Social Skills Rating System Preschool Teacher Form

The validity and reliability studies of the original form of the Social Skills Rating System Preschool Teacher Form (SSRS-PTF), which was developed by Gresham and Elliott (1990) and adapted into Turkish by Aykır and Tekinarslan (2012), were conducted with the data obtained from participants including children with intellectual disabilities in preschool education institutions included in the inclusion program. The SSRS is a behavior rating scale and can be applied to children between the ages of 3–18. The form of the SSRS prepared for the preschool period consists of 40 items and 2 scales. The first scale consisting of the first 30 items is called the Social Skills Scale (SSS), and the scale containing 31 to 40 items is called the Problem Behavior Scale (PBS). The items are graded on a three-point Likert scale as never, sometimes and very often (Aykır & Tekinarslan, 2012).

### Data Analysis

Statistical analysis of the data was performed using SPSS (Statistical Package for the Social Sciences) 25.0 package program. In addition to descriptive statistical methods (Mean, Standard Deviation, Median, Frequency, Ratio, Minimum, Maximum), the Kolmogorov Smirnov test was used to determine whether the data were normally distributed and it was seen that the data were not normally distributed. Data analysis with Mann Whitney U test and Wilcoxon signed-rank test aimed to determine statistically significant differences between the GARS-2 TV, PDMS-2, SSRS-PTF and PASAF test scores obtained before and after 12 weeks of MIP. Mann Whitney U test was used for two-group comparisons of parameters that did not show normal distribution, and Wilcoxon signed-rank test was used to examine the changes between pretest and posttest measurements. Significance was evaluated at p < 0.05 level.

## Findings

### Description of the Participations

In this study, the number of participants was determined by G power analysis. According to the results of the analysis, 34 autistic children were included in the study, 17 in the experimental group (EG) and 17 in the control group (CG). The mean age of EG and CG was 58.05 ± 8.80 months and 56.26 ± 8.97 months, respectively. There was no statistical difference in the mean ages of the two groups (p > 0.05). There were 1 girl and 16 boys in both groups. While the CG only continued their routine program within the special education preschool program, the EG participated in the motor intervention program (MIP) for 60 min a day, 2 days a week for 12 weeks in addition to the routine program (Table 1).

**Table 1:** Description of the participations: gender, mean age and activities carried out by ASD

	EG	CG	p
	Mean ± SD	Mean ± SD	
n	17	17	
Age	58.05 ± 8.80	56.26 ± 8.97	0.352
Gender	1 girl + 16 boys	1 girl + 16 boys	
Activities	MI program for 1 h in 2 days a week for 12 weeks	Continued daily routine special educational activities	

### Effects of MIP on Autistic Index

When the differences between the pretest scores of EG and CG were examined, no statistically significant difference was found in the communication (p = 0.917) and social interaction (p = 0.085) subtests of the GARS-2 TV and in the total standard score (p = 0.172) (p > 0.05). A statistically significant difference was found in the stereotype subtest (p = 0.025; p < 0.05) and it was found to be higher in the CG (Table 2). However, according to the GARS-2 TV, the value that determines the level of autistic index is the total standard score. In this case, although there was a difference in the pre-test scores of the two groups in the stereotype sub-dimension, since there was no difference in the total standard score, we can say that the two groups had similar autistic indexes before MIP. When the GARS-2 TV scores of the two groups were compared again with Mann Whitney U test after MIP, no statistically significant difference was found in the communication subtest (p = 0.577; p > 0.05). However, a statistically significant difference was found in the stereotype (p = 0.004) and social interaction (p = 0.006) subtests and total standard score (p = 0.031) values (p < 0.05) and it was observed that the CG had a higher autistic index (Table 2).

**Table 2:** Comparison of GARS-2-TV Scores of EG and CG

		EG (n = 17)		CG (n = 17)		U	p
		Mean ± SD	Min–Max (Median)	Mean ± SD	Min–Max (Median)		
Pre-test	Stereotype	8.47 ± 2.35	5–13 (8)	10.29 ± 2.2	7–14 (11)	80.5	0.025*
	Communication	8.29 ± 4.31	0–15 (9)	8.12 ± 4.99	0–14 (9)	141.5	0.917
	Social interaction	8.82 ± 2.74	6–15 (8)	9.94 ± 1.95	6–13 (10)	95.0	0.085
	Total standard score	25.59 ± 5.56	19–41 (25)	28.35 ± 6.5	16–39 (29)	105.0	0.172
Post-test	Stereotype	7.88 ± 1.87	5–11 (8)	10.35 ± 2.42	6–14 (10)	60.5	0.004*
	Communication	8.06 ± 4.38	0–13 (10)	8.59 ± 5.37	0–15 (10)	128.5	0.577
	Social interaction	7.71 ± 2.23	4–13 (8)	10.12 ± 2.39	6–14 (10)	65.5	0.006*
	Total standard score	23.65 ± 4.47	18–34 (24)	29.06 ± 7.64	17–41 (28)	82.0	0.031*

Mann Whitney U Test. *p < 0.05

According to the results of the Wilcoxon signed-rank test applied to examine the changes before and after the MIP, a statistically significant difference was found in favor of the participants in the EG in the stereotype (p = 0.026) and social interaction (p = 0.002) subtests and total standard scores (p = 0.001) after the program (p < 0.05). On the other hand, the change in the scores obtained from the communication subtest was not statistically significant (p = 0.279; p > 0.05) (Table 3). In the CG, no statistically significant difference was found in any subtest and total standard score of GARS-2 TV (p > 0.05). These results show that there was a positive change in terms of autistic index values in the EG, but not in the CG (Table 3).

**Table 3:** In-group comparison of GARS-2-TV pre-test and post-test scores

		Pre-test		Post-test		z	p
		Mean ± SD	Min–Max (Median)	Mean ± SD	Min–Max (Median)		
EG (n = 17)	Stereotype	8.47 ± 2.35	5–13 (8)	7.88 ± 1.87	5–11 (8)	− 2.232	0.026*
	Communication	8.29 ± 4.31	0–15 (9)	8.06 ± 4.38	0–13 (10)	− 1.081	0.279
	Social interaction	8.82 ± 2.74	6–15 (8)	7.71 ± 2.23	4–13 (8)	− 3.066	0.002*
	Total standard score	25.59 ± 5.56	19–41 (25)	23.65 ± 4.47	18–34 (24)	− 3.330	0.001*
CG (n = 17)	Stereotype	10.29 ± 2.2	7–14 (11)	10.35 ± 2.42	6–14 (10)	− 0.258	0.796
	Communication	8.12 ± 4.99	0–14 (9)	8.59 ± 5.37	0–15 (10)	− 1.903	0.057
	Social interaction	9.94 ± 1.95	6–13 (10)	10.12 ± 2.39	6–14 (10)	− 0.302	0.763
	Total standard score	28.35 ± 6.5	16–39 (29)	29.06 ± 7.64	17–41 (28)	− 1.161	0.246

Wilcoxon Signed Rank Test. p < 0.05

### Effects of MIP on Pre-Academic Skills

At the beginning of the MIP, there was no statistically significant difference (p > 0.05) between the PASAF scores of EG and CG in terms of color (p = 0.256), geometric shape (p = 0.696), dimension (p = 0.418) and number (p = 0.945) subtests and total score (p = 0.887). This result shows that the participants in the two groups had similar pre-academic skill levels at the beginning of the study. These analyses with Mann Whitney U test showed that there was a statistically significant difference between the PASAF test scores of EG and CG after MIP in terms of color (p = 0.026), dimension (p = 0.002) and number (p = 0.005) subtests and total score (p = 0.004) values in favor of EG (p < 0.05). The posttest scores obtained in the geometric shape subtest revealed that there was no statistically significant difference between the two groups (p > 0.05) (Table 4).

**Table 4:** Comparison of PASAF scores of EG and CG

		EG (n = 17)		CG (n = 17)		U	p
		Mean ± SD	Min–Max (Median)	Mean ± SD	Min–Max (Median)		
Pre-Test	Color	11.47 ± 6.36	3–18 (13)	10.12 ± 6.14	3–18 (9)	112	0.256
	Shape	7.65 ± 4.31	2–12 (6)	7.35 ± 4.57	2–12 (4)	134	0.696
	Dimension	1.29 ± 1.86	0–6 (0)	1.88 ± 2.18	0–6 (2)	123	0.418
	Number	6.94 ± 6.17	0–22 (8)	6.59 ± 5.98	0–17 (4)	142.5	0.945
	Total Score	27.35 ± 17.68	5–58 (26)	25.94 ± 18.22	5–50 (19)	140	0.887
Post-Test	Color	15.24 ± 4.1	8–18 (18)	10.88 ± 6.28	3–18 (10)	84	0.026*
	Shape	9.94 ± 2.97	4–12 (12)	7.59 ± 4.35	2–12 (5)	96	0.064
	Dimension	4.59 ± 1.84	2–6 (6)	2 ± 2.35	0–6 (2)	58	0.002*
	Number	13.35 ± 6.08	4–22 (15)	6.94 ± 5.95	0–17 (5)	64	0.005*
	Total Score	43.12 ± 14.5	21–58 (51)	27.41 ± 18.29	6–53 (21)	60.5	0.004*

Mann Whitney U Test. *p < 0.05

According to the analyses performed with the Wilcoxon signed-rank test, there was a statistically significant difference between the pre-test and post-test scores of EG in all subtests and total score values in the preliminary academic skills (p < 0.05). In CG, no statistically significant difference was found in the geometric shape (p = 0.102) and dimension (p = 0.317) subtests (p > 0.05), whereas a statistically significant difference was found in the color (p = 0.004) and number (p = 0.034) subtests and total score (p = 0.001) (p < 0.05). This change in CG can be explained by the fact that these children routinely attended preschool special education programs. These results showed that the pre-academic skill levels of the EG who participated in the program increased in all subtests and total scores, and that EG had higher mean scores in all subtests and total scores, despite the change seen in the color and number subtests and total score in the CG (Table 5).

**Table 5:** In-group comparison of pre-test and post-test scores of PASAF

		Pre-test		Post-test		z	P
		Mean ± SD	Min–Max (Median)	Mean ± SD	Min–Max (Median)		
EG (n = 17)	Color	11.47 ± 6.36	3–18 (13)	15.24 ± 4.1	8–18 (18)	− 2.814	0.005*
	Shape	7.65 ± 4.31	2–12 (6)	9.94 ± 2.97	4–12 (12)	− 2.680	0.007*
	Dimension	1.29 ± 1.86	0–6 (0)	4.59 ± 1.84	2–6 (6)	− 3.573	0.000*
	Number	6.94 ± 6.17	0–22 (8)	13.35 ± 6.08	4–22 (15)	− 3.530	0.000*
	Total Score	27.35 ± 17.68	5–58 (26)	43.12 ± 14.5	21–58 (51)	− 3.519	0.000*
CG (n = 17)	Color	10.12 ± 6.14	3–18 (9)	10.88 ± 6.28	3–18 (10)	− 2.919	0.004*
	Shape	7.35 ± 4.57	2–12 (4)	7.59 ± 4.35	2–12 (5)	− 1.633	0.102
	Dimension	1.88 ± 2.18	0–6 (2)	2.00 ± 2.35	0–6 (2)	− 1.000	0.317
	Number	6.59 ± 5.98	0–17 (4)	6.94 ± 5.95	0–17 (5)	− 2.121	0.034*
	Total Score	25.94 ± 18.22	5–50 (19)	27.41 ± 18.29	6–53 (21)	− 3.222	0.001*

Wilcoxon Signed Rank Test. p < 0.05

### Effects of MIP on Social Skills

In this study, when the pre-test scores of EG and CG were compared, no statistically significant difference was found between the two groups in all sub-dimensions including cooperation (p = 0.986), self-expression (p = 0.446), self-control (p = 0.305), externalized behaviors (p = 0.553) and internalized behaviors (p = 0.815) (p > 0.05). After the program was completed, while there was no difference in the sub-dimensions of self-expression and self-control (p > 0.05), a statistically significant difference was found in favor of EG in the sub-dimensions of cooperation (p = 0.028), externalized behaviors (p = 0.016) and internalized behaviors (p = 0.009) (p < 0.05). These results showed that before the MIP, EG and CG were similar in terms of social skills levels, but after the program, there was a change in favor of EG in the 3 subtests of the Social Skills Rating System Preschool Teacher Form (SSRS-PTF) (Table 6).

**Table 6:** Comparison of SSRS-PTF scores of EG and CG

		EG (n = 17)		CG (n = 17)		U	p
		Mean ± SD	Min–Max (Median)	Mean ± SD	Min–Max (Median)		
Pre-Test	Collaboration	0.55 ± 0.33	0–1.14 (0.64)	0.57 ± 0.37	0.21–1.35 (0.5)	144	0.986
	Self-expression	0.58 ± 0.40	0–1.25 (0.62)	0.66 ± 0.32	0.25–1.37 (0.5)	122.5	0.446
	Self-control	0.67 ± 0.4	0–1.5 (0.62)	0.84 ± 0.36	0.5–1.75 (0.75)	115	0.305
	Externalizing	0.68 ± 0.40	0.16–1.33 (0.5)	0.74 ± 0.37	0.16–1.33 (0.66)	127	0.553
	Internalizing	0.42 ± 0.29	0–1 (0.5)	0.44 ± 0.24	0–1.75 (0.50)	138	0.815
Post-Test	Collaboration	0.88 ± 0.41	0.21–1.71 (0.85)	0.55 ± 0.40	0.07–1.42 (0.42)	81	0.028*
	Self-expression	0.73 ± 0.44	0.12–1.62 (0.62)	0.63 ± 0.31	0.12–1.25 (0.62)	131	0.639
	Self-control	0.94 ± 0.43	0.37–2 (0.87)	0.81 ± 0.30	0.5–1.50 (0.75)	124	0.475
	Externalizing	0.45 ± 0.30	0–1 (0.5)	0.81 ± 0.42	0.16–1.50 (0.66)	75	0.016*
	Internalizing	0.29 ± 0.23	0–0.50 (0.50)	0.54 ± 0.25	0–1 (0.50)	74.5	0.009*

Mann Whitney U Test. *p < 0.05

When the scores of EG obtained before and after MIP were compared, a statistically significant difference (p < 0.05) was found in the sub-dimensions of cooperation (p = 0.001), self-control (p = 0.012), externalized behaviors (p = 0.003) and internalized behaviors (p = 0.013), while no difference was found in the sub-dimension of self-expression (p = 0.208) (p > 0.05). In CG, no statistically significant difference was observed in any sub-dimension (p > 0.05). These results obtained with the Wilcoxon signed-rank test revealed that autistic children in EG had an increase in social skills levels at the end of MIP (Table 7).

**Table 7:** In-group comparison of pre-test and post-test scores of SSRS-PTF

		Pre-Test		Post-Test		z	p
		Mean ± SD	Min–Max (Median)	Mean ± SD	Min–Max (Median)		
EG (n = 17)	Collaboration	0.55 ± 0.33	0–1.14 (0.64)	0.88 ± 0.41	0.21–1.71 (0.85)	− 3.271	0.001*
	Self-expression	0.58 ± 0.40	0–1.25 (0.62)	0.73 ± 0.44	0.12–1.62 (0.62)	− 1.260	0.208
	Self-control	0.67 ± 0.4	0–1.5 (0.62)	0.94 ± 0.43	0.37–2 (0.87)	− 2.523	0.012*
	Externalizing	0.68 ± 0.40	0.16–1.33 (0.5)	0.45 ± 0.30	0–1 (0.5)	− 2.974	0.003*
	Internalizing	0.42 ± 0.29	0–1 (0.5)	0.29 ± 0.23	0–0.50 (0.50)	− 2.496	0.013*
CG (n = 17)	Collaboration	0.57 ± 0.37	0.21–1.35 (0.5)	0.55 ± 0.40	0.07–1.42 (0.42)	− 0.355	0.722
	Self-expression	0.66 ± 0.32	0.25–1.37 (0.5)	0.63 ± 0.31	0.12–1.25 (0.62)	− 0.554	0.579
	Self-control	0.84 ± 0.36	0.5–1.75 (0.75)	0.81 ± 0.30	0.5–1.50 (0.75)	− 1.190	0.234
	Externalizing	0.74 ± 0.37	0.16–1.33 (0.66)	0.81 ± 0.42	0.16–1.50 (0.66)	− 0.861	0.389
	Internalizing	0.44 ± 0.24	0–1.75 (0.50)	0.54 ± 0.25	0–1 (0.50)	− 1.706	0.088

Wilcoxon Signed Rank Test. p < 0.05

### Effects of MIP on Motor Skills

When the Peabody motor development scale 2 (PDMS-2) scores of EG and CG before MIP were compared, no statistically significant difference was observed between the two groups in terms of balance skills (p = 0.142), locomotor skills (p = 0.143), manipulative skills (p = 0.057) and total gross motor (p = 0.129) scores (p > 0.05). At the end of MIP, a statistically significant difference was found in favor of EG in terms of all subtests and total gross motor score (p < 0.05). According to the Mann Whitney U test, while the two groups had similar motor skill levels at the beginning of the study, there was an increase in the motor skill levels of EG after MIP (Table 8).

**Table 8:** Comparison of pre-test and post-test scores of EG and CG on PDMS-2

		EG (n = 17)		CG (n = 17)		U	p
		Mean ± SD	Min–Max (Median)	Mean ± SD	Min–Max (Median)		
Pre-Test	Balance	49.18 ± 4.5	40–55 (49)	46.53 ± 5.98	40–56 (46)	102	0.142
	Locomotor	146.35 ± 15.17	112–165 (149)	136.29 ± 18.42	112–164 (132)	102	0.143
	Manipulative	35.71 ± 5.41	21–42 (37)	32.82 ± 4.88	26–41 (33)	89.5	0.057
	Total gross motor score	231.24 ± 23.95	173–256 (238)	215.65 ± 28.30	182–261 (213)	100.5	0.129
Post-Test	Balance	55.94 ± 3.98	45–60 (57)	47.65 ± 6.34	40–58 (46)	45	0.001*
	Locomotor	164.65 ± 12.12	126–177 (168)	137.41 ± 18.31	116–168 (134)	29	0.000*
	Manipulative	42.41 ± 4.99	28–47 (43)	33.35 ± 5.09	25–42 (33)	29	0.000*
	Total gross motor score	263.00 ± 20.55	199–283 (266)	218.41 ± 28.59	185–266 (218)	29.5	0.000*

Mann Whitney U Test. *p < 0.05

According to the results of Wilcoxon signed-rank test analysis, the difference between the scores obtained before and after MIP for balance skills (p = 0.001), locomotor skills (p = 0.001), manipulative skills (p = 0.001) and total gross motor skills (p = 0.000) in EG was statistically significant (p < 0.05). Except for manipulative skills (p = 0.070; p > 0.05), there was a statistically significant difference (p < 0.05) in the increase observed in the other subtests and total gross motor skill score of the CG. According to these results, it was determined that there was improvement in the motor skill levels of the participants in both groups; however, it was found that the EG with MIP had higher mean scores than the CG in all subtests and total gross motor skill scores (Table 9).

**Table 9:** In-group comparison of PDMS-2 pre-test and post-test scores

		Pre-Test		Post-Test		z	p
		Mean ± SD	Min–Max (Median)	Mean ± SD	Min–Max (Median)		
EG (n = 17)	Balance	49.18 ± 4.5	40–55 (49)	55.94 ± 3.98	45–60 (57)	− 3.653	0.001*
	Locomotor	146.35 ± 15.17	112–165 (149)	164.65 ± 12.12	126–177 (168)	− 3.628	0.001*
	Manipulative	35.71 ± 5.41	21–42 (37)	42.41 ± 4.99	28–47 (43)	− 3.636	0.001*
	Total gross motor score	231.24 ± 23.95	173–256 (238)	263.00 ± 20.55	199–283 (266)	− 3.622	0.000*
CG (n = 17)	Balance	46.53 ± 5.98	40–56 (46)	47.65 ± 6.34	40–58 (46)	− 2.796	0.005*
	Locomotor	136.29 ± 18.42	112–164 (132)	137.41 ± 18.31	116–168 (134)	− 2.355	0.019*
	Manipulative	32.82 ± 4.88	26–41 (33)	33.35 ± 5.09	25–42 (33)	− 1.812	0.070
	Total gross motor score	215.65 ± 28.30	182–261 (213)	218.41 ± 28.59	185–266 (218)	− 3.399	0.001*

Wilcoxon Signed Rank Test. p < 0.05

## Discussion

This study revealed that motor intervention program** (**MIP) applied to children on the autism spectrum had a positive effect on motor skills, pre-academic skills, social skills and autistic index. The results of the study, which are discussed together with the results of the literature on the subject, are presented below.

### Description of the Participations

This study was conducted with a total of 34 autistic children, 17 in the experimental group (EG) and 17 in the control group (CG). There was no statistical difference between the mean ages of the EG (58.05 ± 8.80) and the CG (56.26 ± 8.97) (p > 0.05). While CG only continued their routine program within the special education preschool program, DG participated in the motor intervention program (MIP) for 60 min a day, 2 days a week for 12 weeks in addition to the routine program (Table 1).

### Effects of MIP on Autistic Index

Recent studies focusing on preschool autistic children emphasize that early motor interventions will have a positive impact on the characteristics of autism (Chu & Pan, 2012; Hilton et al., 2007; Kaur et al., 2018; Keskin et al., 2017; MacDonald et al., 2014; McLaughin 2010; Obrusnikova & Miccinello, 2012; Öz, 2021; Prupas & Reid, 2001; Richmond, 2000; Sedehi et al., 2021). In one of these studies, after 15 min of different forms of physical activity (walking, running and ball throwing, etc.), the stereotypic activity levels of the participants were compared with their baseline levels after each exercise and the frequency of stereotypic behaviors was recorded for 3 weeks. Looking at the averages of the three participants, it was reported that the greatest decrease in stereotypic behaviors was seen after running exercises, followed by ball throwing and walking exercises, respectively (Richmond, 2000).

McLaughin (2010), in his study on three male autistic children aged 3–5 years, obtained similar results showing that an intensive physical activity program reduced stereotypic behaviors. In another study in the literature, it was reported that kata techniques training applied for 14 weeks had an effect that significantly reduced stereotypic behaviors in autistic children and the reduced stereotypic movements of the participants were maintained even 30 days after the end of the interventions (Bahrami et al., 2012). In a study by Öz (2021), it was reported that the gamified physical activity program applied to autistic children for 14 days had a positive effect on social interaction and stereotype behaviors. Chu and Pan (2012) found that teacher-guided, peer-supported and volunteer-led in-water exercises improved the social interaction behaviors of autistic children.

Keskin et al. (2017) reported that different exercise interventions had positive effects on motor performance as well as stereotypic behavior rates in autistic children aged 5–8 years. In the same study, it was also reported that children's interactions with their parents in the home environment, with special education teachers during individual trainings, and with the tools and materials around them increased (Keskin et al., 2017). In another study conducted with 4 autistic children between the ages of 5–9, participants were given an exercise program consisting of 10 min of light jogging in the company of typically developing peers. According to the findings of the study, the reduction in stereotypic behaviors was 51.6% when the exercises were performed once a day and 58.9% when the exercises were performed 3 times a day (Prupas & Reid, 2001). These results suggest that the frequency and intensity of exercise are also important on the effect of interventions applied to autistic children.

The findings obtained from our study are in parallel with the findings of the literature mentioned above. In our study, it was found that there was a positive decrease in the stereotype and social interaction sub-dimensions and standard score values of EG after MIP. Therefore, it was concluded that MIP provided a positive change in the autistic index level of the EG. However, the change in communication sub-dimension scores was not significant (Table 3). Communication skills are one of the most negatively affected developmental areas of autistic children (Diken, 2014). Most of these children have difficulty in understanding small differences or relationships in facial expressions and body movements. Stress, lack of familiarity with the environment or the inability to share thoughts in a one-way or reciprocal way cause them to experience inadequacies in social communication and interaction (Grenier, 2013).

Although there was an increase in the mean scores of EG in the communication sub-dimension, no statistical difference was found. When this result is evaluated considering the age of the research group, it suggests that this age group needs more intensive and long-term support in terms of communication skills. On the other hand, the positive change in the stereotype and social interaction sub-dimensions as well as the total standard scores determining the level of autistic index clearly shows that MIP has a positive effect on autism traits.

### Effects of MIP on Pre-Academic Skills

Children are generally very mobile and are constantly under the influence of a play-movement and displacement drive. Body movements are undoubtedly the most important of the experiences necessary for the enrichment of the complex brain structure with the experiences to be acquired after birth. Body movements are the most direct way of communicating with the world and experiencing it. Children are born structured in such a way that they can use this opportunity in abundance (Canan, 2019).

Precisely for these reasons, it is known that children can quickly lose their excitement and interest in activities that are carried out in a calm environment, away from movement and in a fixed manner in the preschool period. On the other hand, by utilizing children's interest in play, pre-academic skills can be acquired in a fun way in play-based teaching environments (Uyanık & Kandır, 2010). Moreover, movement-based practices can offer educators different opportunities in terms of teaching strategies to reinforce academic concepts, develop problem-solving skills, and support critical thinking skills in children. Robert Sylwester, author of the book "Celebration of neurons", states that the experiences gained through movement-based practices contribute to an individual's strong memory and learning ability (as cited in Özer & Özer, 2012).

In one study, the changes in the academic skills of 10 autistic children aged 5–16 years participating in an inclusive physical activity program were examined before and after starting sports, and it was reported that all participants had positive improvements in their ability to use measurements, rhythmic counting, understanding natural numbers and distinguishing geometric shapes after sports practices (Parlak, 2021). The results of a meta-analysis study conducted by De Greeff et al. (2018) explained that physical activity has positive effects on executive functions, attention and academic performance in children on the autism spectrum. Fulceri et al. (2015) reported that motor skills were significantly related to non-verbal IQ and adaptive behaviors, and that children with poorer motor skills had more cognitive and adaptive behavior deficits.

Studies showing a strong relationship between early gross motor development and cognitive development at school age draw attention to the fact that early locomotor experiences are an important factor for developmental change and the critical importance of appropriate interventions in the early period (Campos et al., 2000; Piek et al., 2008). These findings emphasize the increasing importance of motor skills in terms of social development, cognitive development and communication in later years and reveal that motor programs are a key area in the special education and rehabilitation of preschool autistic children.

Our research findings revealing positive changes in EG after MIP support the aforementioned research results. However, another conclusion reached through this research is that there is an increase in CG's PASAF scores. In addition, a statistically significant difference was found in favor of EG in color, size and number subtests and total score values in terms of post-test scores of PASAF. Although there was no statistically significant difference in the Figure subtest, there was a remarkable increase in the EG scores.

Within the scope of the preschool special education curriculum in Turkey, a curriculum that includes colors, numbers and geometric shapes under the title of "cognitive development" is implemented in line with the goals set for developmental areas (Milli Eğitim Bakanlığı [Turkish Ministry of National Education], 2018). All participants of the study attend a preschool special education program. Despite the change in the increase in the scores of CG, which we think that the educational process they received within the scope of special education had an effect, the fact that the scores obtained were higher in favor of EG in all subtests and total scores reveals the effect of the MIP we applied.

### Effects of MIP on Social Skills

Profound difficulties in social functioning are one of the defining features of autism. Even though many autistic children want to interact with others, they often lack the skills necessary to carry out social exchanges effectively. Despite this, many early intervention programs focus primarily on teaching pre-academic skills. However, it is not enough for autistic children to be in physical proximity with their peers or to be limited to interventions that focus on teaching pre-academic skills to close social skills gaps (Howell, 1985; as cited in Scattone, 2007).

Recent studies have highlighted evidence that motor programs have significant effects on social skill development as well as motor skill development in autistic children (Alexander et al., 2011; Bremer et al., 2015; Chu & Pan, 2012; Esentürk, 2019; Ketcheson et al., 2017; Orhan, 2020; Parlak, 2021; Sansi et al., 2021; Sowa & Meulenbroek, 2012). In one of these studies by Ketcheson et al. (2017), an intensive motor support program with peer participation was implemented for 4 h a day, 5 days a week for autistic children aged 4–6 years, and it was reported that the program had positive effects on the development of social and motor skills. The study also emphasized the importance of including motor programming as part of early intervention services for young children (Ketcheson et al., 2017). In another study with similar results, it was reported that autistic children participating in the Inclusive Adapted Physical Activity program increased their level of interaction with their peers, improved their leisure time skills, and had significant changes in their social and communication skills at the end of the program (Sansi et al., 2021).

Bremer et al. (2015) also draw attention to similar results that exercise practices have a significant effect on adaptive behaviors and social skills. The results of another study examining the changes in problem behaviors of a 3-year-old autistic child who participated in adapted physical activities reported a significant decrease in behavioral problems, externalized problems and internalized problems of the participant after the activities (Orhan, 2020). Many other studies examining the effects of exercise and physical activity-based programs on different variables have shown that these programs have positive effects on autistic children's social communication and interactions (Derer, 2018; Esentürk, 2019; Yanardağ et al, 2009), children were more willing to participate in games, started to spend more time with their peers, increased their verbal communication, showed positive progress in taking commands, following instructions and rules (Parlak, 2021), and even made significant improvements in eye contact as well as social skills development (Alexander et al., 2011).

Although autistic children have the basic skills necessary for socialization, they may not be able to fulfill these skills because they are not interested in social interaction (Durand, 2005). However, with well-planned motor programs, children can develop the skills to move and cooperate in harmony with other children (Özer & Özer, 2012). Therefore, it is important to give these children the chance to be a part of early intervention programs, to develop motor skills that will enable them to be involved in the process, and to provide opportunities to support their communication and social skills that can enable them to take part with their peers in educational and recreational environments (Duronjić & Válková, 2010).

Our findings in this study, which are consistent with the literature, show that MIP has positive effects on cooperation (following instructions, participating in games and group activities, etc.), self-control (waiting for one's turn, responding positively to criticism, etc.), externalized behaviors (hyperactivity, maladaptive behaviors, not following rules, etc.), and internalized behaviors (appearing alone, anxiety about playing with a group of children, etc.), but the change in the self-expression sub-dimension was insignificant. Our findings also show that there was no change in CG (Table 7). These results suggest that MIP functions as a kind of laboratory for autistic children to improve their social skills.

### Effects of MIP on Motor Skills

Research on the motor development of autistic young children points to significant motor delays and that delays become more pronounced with age (Elliott et al., 2021; Kruger et al., 2019; Landa & Garrett-Mayer, 2006; Lloyd et al., 2013; Odeh et al., 2022; Oliveira et al., 2021; Özcan et al., 2023). Motor delays in ASD can have a negative impact on language development, social development and cognitive development. Failure to assess and treat motor delays can make it difficult to address other common developmental difficulties in autistic children (Liu & Breslin, 2013). Despite this, motor skills are neglected in early childhood education (Lloyd et al., 2013) and supporting motor difficulties is not a priority for parents (Redquest et al., 2020). However, development is a whole and neglecting a part of an autistic child's development is like leaving the soil without water and it should not be forgotten that a young child pays the price (Özcan et al., 2023).

In this study, which we set out with the belief that delays in motor skills of preschool autistic children should be supported in a way to include other developmental areas, a significant increase was observed in balance, locomotor and manipulative skills and total gross motor scores of EG after MIP (Table 8). Similar to our study, Bremer et al. (2015) reported that the motor support program they applied to 4-year-old autistic children for 12 weeks provided significant gains in all subtests of Peabody motor development scale 2 (PDMS-2) and total gross motor skills. Another study conducted with autistic children aged 4–6 years showed statistically significant results in favor of the experimental group in terms of locomotor skills, object control skills and total gross motor scores (Ketcheson et al., 2017).

Sarabzadeh et al. (2019) applied 6-week Tai Chi Chuan training to 18 children aged 6–12 years on the autism spectrum and found an increase in ball skills and balance performance subtests in the experimental group. Another study examining similar effects reported improvements in both motor skill proficiency and social skills in autistic children aged 3–7 years who participated in 6-week basic motor skills training (Bremer & Lloyd, 2016). For autistic children and infants at risk for ASD, the importance of timely comprehensive motor assessments and addressing motor impairments with effective interventions is clear. Therefore, there is an urgent need to develop embodied strategies based on movement and motor learning principles (Bhat et al., 2011).

In this study, in which we also examined the differences between the in-group pre-test scores and post-test scores of EG and CG in terms of PDMS-2, it was found that the increase in the post-test scores of EG in balance, locomotor and manipulative subtests and total gross motor was statistically significant. A statistically significant difference was found in balance, locomotor subtests and total gross motor scores of CG, but the change in the manipulative subtest score was not statistically significant. Despite the increase in balance and locomotor scores and total gross motor score in CG, it was concluded that the mean values obtained in all subtests and total score were much higher in favor of EG.

The period of basic movements between the ages of 2–7 is a period in which the child rapidly climbs to the top developmentally and major changes are observed. In this period, motor skills increase in terms of maturation and quality with age (Garcia & Garcia, 2006; Goran, 1998; Moore et al., 2003). For this reason, it is reported that some children reach the maturity level of basic movement skills despite the minimum level of environmental factors (Goodway et al., 2019). Based on this information, the increase observed in the balance and locomotor subtests and total gross motor scores of the CG, despite not participating in the MIP, can be explained by the knowledge that motor skills in the preschool period can develop to a certain extent depending on age. In the end, although there was an increase in the motor skill levels of the CG, the mean scores of the EG in all subtests of the PGMS-2 and total gross motor skill were significantly higher than the CG (Table 9).

Research on the subject reveals the gains of motor programs on secondary effects such as play skills, daily living skills, social interaction and communication skills, and academic skills depending on the development of motor skills of autistic children. In fact, Elliott et al. (2021) reported that the benefits of early motor skills programs are not only limited to motor development, but also extend to family members and thus positively affect family dynamics. All these results suggest that motor programs improve the quality of life of autistic children by supporting various developmental areas. This situation reveals the importance of accepting these programs as a fundamental element in the education of autistic children.

## Limitations

In the literature, there are very few studies examining the autistic index, motor skills, social skills and pre-academic skills of autistic preschool children. Therefore, the results of this study were compared with the limited number of studies available in the literature.

In this study, the opinions of parents and special education teachers on the effects of the program could have been obtained and their awareness on the subject could have been examined. However, due to the current conditions, this study was limited to the use of quantitative research methods and techniques. In future studies, a mixed methodology using both quantitative and qualitative data may be preferred in order to examine the subject in a more comprehensive and detailed manner.

Approximately 1/3 of the participants in this study left the institutions where they received training for various reasons shortly after the last measurements were completed. Therefore, another limitation of this study is that we could not conduct the follow-up tests that we planned to repeat in order to examine the long-term effects of motor intervention program.

## Conclusion

In this study, it was concluded that the 12-week motor intervention program (MIP) was an effective method to improve motor skills, social skills and pre-academic skills of autistic toddlers and positively reduced the autistic index levels of autistic children who participated in the program. In this context, this study reveals that motor programs structured to include other developmental domains provide a conducive environment for autistic children to develop multiple skills.

It is known that motor skill-focused trainings for autistic children contribute when implemented directly and within an intensive program. In addition, the importance of offering these programs especially during the critical period of 0–6 years is clear (Busti Ceccarelli et al., 2020; Colombo-Dougovita & Block, 2019; Elliott et al., 2021; Ketcheson et al., 2017; Piek et al., 2008). For these reasons, supporting multiple skills in the same time period in the early period of life will provide maximum learning opportunities for children on the autism spectrum by creating a learning environment with rich stimuli. Moreover, these results support the widespread use of motor programs as an alternative practice to address parents' and teachers' concerns about social and academic deficits (Kurtz, 2007; Lloyd et al., 2013; Redquest et al., 2020).

The evaluations conducted to better understand the child serve as a guide in determining the right strategies for the child's educational needs and taking the necessary measures to ensure maximum benefit from the child's educational process. For this reason, it is thought that the participant monitoring form, in which the child's interest in the education program, performance and positive or negative reactions to the activities during the training session were recorded at the end of each training session, contributed to the effectiveness of the MIP.

There is a need for more studies to reveal the effects of motor skill-focused programs to be implemented in early childhood, taking into account the diversity of skills within the scope of developmental areas. Nevertheless, while MIP appears to be effective in the short term (for a 12-week period), longer-term benefits beyond 12 weeks need to be assessed.
